# Patent infections with soil-transmitted helminths and *Schistosoma mansoni* are not associated with increased prevalence of antibodies to the *Onchocerca volvulus* peptide epitopes OvMP-1 and OvMP-23

**DOI:** 10.1186/s13071-019-3308-z

**Published:** 2019-01-28

**Authors:** Johnny Vlaminck, Ole Lagatie, Ann Verheyen, Daniel Dana, Bieke Van Dorst, Zeleke Mekonnen, Bruno Levecke, Lieven J. Stuyver

**Affiliations:** 1 Department of Virology, Parasitology and Immunology, University of Ghent, Salisburylaan 133, 9820 Merelbeke, Belgium; 2 Janssen Diagnostics, Janssen R&D, Turnhoutseweg 30, 2340 Beerse, Belgium; 3 School of Medical Laboratory Sciences, Jimma University, Jimma, Ethiopia

**Keywords:** *Onchocerca volvulus*, River blindness, Onchocerciasis, Serology, Linear epitope, Soil-transmitted helminths

## Abstract

**Background:**

Ov16 serology is considered a reference method for *Onchocerca volvulus* epidemiological mapping. Given the suboptimal sensitivity of this test and the fact that seroconversion takes more than a year after infection, additional serological tests might be needed to guide onchocerciasis elimination programmes. Recently, two linear epitopes encoded in OvMP-1 and OvMP-23 peptides were introduced as serological markers, but the observed antibody cross-reactivity in samples originating from *Onchocerca volvulus* non-endemic areas required further investigation.

**Methods:**

We evaluated both peptide markers in an *O. volvulus* hypo-endemic setting in Jimma Town, Ethiopia using peptide ELISA. For all individuals (*n* = 303), the infection status with soil-transmitted helminths and *Schistosoma mansoni* was known.

**Results:**

We found that 11 (3.6%) individuals were positive for anti-Ov16 IgG4 antibodies, while 34 (11.2%) and 15 (5.0%) individuals were positive for OvMP-1 and OvMP-23, respectively. Out of the 34 OvMP-1 positive samples, 33 were negative on the Ov16 IgG4 ELISA. Similarly, out of the 15 OvMP-23 positive samples, 14 scored negative on this reference method. No difference in seroprevalence for all three markers could be observed between uninfected individuals and individuals infected with different soil-transmitted helminths or *S. mansoni*. Moreover, the intensity of the response to OvMP-1, OvMP-23 or Ov16 was not significantly stronger in individuals carrying patent STH or *S. mansoni* infections, nor was there any correlation between the intensities of the responses to the three different antigens.

**Conclusions:**

This study demonstrates that a patent infection with either soil-transmitted helminths or *S. mansoni* does not lead to increased antibody recognition of both OvMP-1 and OvMP23.

**Electronic supplementary material:**

The online version of this article (10.1186/s13071-019-3308-z) contains supplementary material, which is available to authorized users.

## Background

Onchocerciasis (river blindness) is one of the 20 debilitating neglected tropical diseases (NTDs) that have been listed by the World Health Organization (WHO) [1–3]. Onchocerciasis is an eye and skin disease caused by infection with the filarial worm *Onchocerca volvulus*. Worldwide there are 198 million people at risk of onchocerciasis, of which 99% live in Africa. In 2012 it was estimated that 17 million people are affected by the disease or at risk of infection in Ethiopia, making it one of the most affected countries worldwide [4]. In 2013, the Ethiopian government launched the Onchocerciasis Elimination Programme aimed at nationwide interruption of transmission of the disease by 2020 [5]. This programme is based on biannual mass drug administration (MDA) of the microfilaricidal agent ivermectin (Mectizan, Merck & Co., Inc., Kenilworth, New Jersey, USA).

One of the largest epidemiological mapping efforts for *O. volvulus* was conducted in 20 African countries, called Rapid Epidemiological Mapping of Onchocerciasis (REMO) in support of the African Programme for Onchocerciasis Control (APOC). The main objective of REMO was to identify all high-risk areas where ivermectin treatment was needed. For this programme, diagnosis was based on examination of 30 to 50 adults for the presence of palpable onchocercal nodules in selected villages [6, 7]. Besides the detection of palpable nodules and presence of microfilariae in skin biopsies, the most widely used test for monitoring and evaluation of MDA programmes currently is the detection of IgG4 antibodies to the parasitic antigen Ov16 [8–15]. Although such antibody test cannot distinguish between past and current infections, the presence of anti-Ov16 antibodies in young children provides evidence for recent exposure [8]. Several studies have shown that Ov16 IgG4 testing is useful for assessing ongoing transmission of onchocerciasis following MDA in Latin America and Africa [15]. However, although Ov16 IgG4 serology has excellent specificity, it appears to have only moderate sensitivity. Sensitivity further decreases when the rapid diagnostic test (RDT) for the detection of Ov16 IgG4 antibodies is used [8, 9, 11]. Discussions are ongoing about the threshold that should be used to determine when it is safe to stop MDA based on Ov16 seroprevalence [16, 17]. Current guidelines indicate 0.1% Ov16 serology in children under 10 years of age, but this is neither practical nor possible with the current Ov16 based tools as even a specificity of 97–98% is not sufficient to enable reliable detection of < 0.1% prevalence [10, 11].

Recently, two peptide-based serology markers (OvMP-1 and OvMP-23) were described and their diagnostic performance evaluated [18]. Both peptides showed high diagnostic sensitivity (100% and 92.7%, respectively) and specificity (98.7% and 100%, respectively). Neither of these peptides showed significant cross-reactivity in sera from *Wuchereria bancrofti-*infected individuals. However, especially for peptide OvMP-1, substantial reactivity was detected in samples originating from Indonesian individuals infected with *Brugia malayi* or soil-transmitted helminths (STHs). Due to the small sample set (*B. malayi*-infected: *n* = 20; STH-infected: *n* = 20) and the limited background information available on these individuals, a more thorough investigation into the cross-reactivity of these diagnostic peptides in *Onchocerca* endemic and non-endemic settings towards STH infections was necessary.

The development of newly discovered biomarkers as diagnostic tools depends largely on the proven clinical utility of these biomarkers. In the first phase of biomarker validation, the analytical validation, it is key to determine the sensitivity and specificity of a biomarker. Therefore, biobanks containing samples from clear-cut cases and controls should be obtained and evaluated. Additionally, especially in the field of infectious diseases, there is a need to confirm that the biomarker is not affected by closely related conditions. In the case of biomarkers for onchocerciasis, it is of absolute importance to evaluate novel biomarkers in individuals that are infected with other helminths, such as *W. bancrofti* and *B. malayi*, causing lymphatic filariasis (LF), soil-transmitted helminths (STHs) or schistosomes, but live in non-, hypo- or meso-endemic areas for *Onchocerca* (0%, < 20%, and between 20–45% nodule prevalence in adult males, respectively [6]). In this study, we specifically evaluated the reactivity towards the serological markers OvMP-1 and OvMP-23 in an area in Ethiopia that is highly endemic for STH and *S. mansoni*, but hypo-endemic for *Onchocerca.*

## Methods

### Study site and study population

The samples used in this study originated from the population of Jimma Town, south-west Ethiopia and were originally collected as part of a study focused on STH diagnostics (Dana et al*.*, unpublished data). Jimma Town is considered a hypo-endemic area for *O. volvulus*, but with moderate to high infection rates for STHs and *S. mansoni* [19–22]. Although the prevalence of onchocerciasis in the population of Jimma Town itself is generally very low, MDA with ivermectin is ongoing since 2014. Moreover, Jimma Town is non-endemic for other filarial infections including *Mansonella perstans* [23], *Loa loa* [24] or *Wuchereria bancrofti* [4, 25]. Study participants were school-aged children (aged 5 to 18 years) and adults (18 to 70 years-old) living within the city limits of Jimma Town.

### Sample collection

The participants of the study were asked to provide a single stool sample of at least 5 g of stool in a clean, labeled stool container. To limit the number of false negative samples, all samples were processed using the Kato-Katz thick smear (0.0417 g), Mini-FLOTAC (2 g of stool) and McMaster egg counting method (2 g of stool) for the detection and enumeration of STH and *S. mansoni* eggs. Individuals were considered to be infected with STHs or *S. mansoni* if any of the three coprological techniques showed the presence of worm eggs. Individuals that were positive for more than one helminth species were categorized as having a mixed infection. An overview of the number of samples distributed by age and helminth infection is provided in Table 1. In addition, 2 ml of venous blood was collected and following centrifugation, serum samples were separated and stored at -20 °C before shipping to the laboratory of parasitology of Ghent University, Belgium for ELISA evaluation. All collected coprological and serological data of all evaluated samples are available in Additional file 1: Table S1.

**Table 1:** Age category and STH infection status of study population used in this study

Age group (years)	*n*	*A. lumbricoides* infected (*n*)	*T. trichiura* infected (*n*)	Hookworm infected (*n*)	*S. mansoni* infected (*n*)	Mixed infection (*n*)	Absence of patent infection (*n*)
< 10	87	17	20	3	2	25	20
14–17	86	8	22	5	1	27	23
18–24	50	10	4	6	2	14	14
> 24	80	12	15	1	0	13	39
Total	303	47	61	15	5	79	96

### Total IgG peptide ELISA for OvMP-1 and OvMP-23

C-terminally biotinylated synthetic peptides OvMP-1 (VSV-EPVTTQET-VSV) and OvMP-23 (VSV-KDGEDK-VSV-QTSNLD-VSV) were synthesized by standard procedures and purchased from PEPperPRINT GmbH (Heidelberg, Germany). For determination of peptide specific serum antibody levels, a peptide ELISA was developed and set up as described previously [18, 26]. The cut-offs for OvMP-1 and OvMP-23 were previously determined and were set at background-corrected OD values of 0.045 and 0.110, respectively [18].

### Ov16 IgG4 ELISA

Recombinant *O. volvulus* Ov16 antigen was purchased from Cusabio Biotech Co., Ltd (College Park, MD, USA) and dissolved in water at a concentration of 1 mg/ml. For determination of Ov16 specific IgG4 levels, an ELISA was developed and set up as follows. Maxisorp 96-well plates were incubated overnight at 4 °C with 100 μl of Ov16 antigen, diluted at 1 μg/ml in PBS. The plates were rinsed once with 300 μl PBS + 0.05% Tween-20 (washing buffer), before being blocked with 300 μl of Superblock™ Blocking Buffer (Thermo Fisher Scientific, Breda, the Netherlands) for 1 h at room temperature. The plates were rinsed 3 times with washing buffer. Then, the different wells were covered with 100 μl of human serum samples, diluted 200-fold in Blocking Buffer. In “blank” control wells, Blocking Buffer was added instead. The plate was incubated at room temperature for 1 h. After incubation, a 5-fold rinsing cycle with washing buffer was performed. Then, the secondary antibody solution was added to each well. The solution contained a mouse monoclonal HP6025 Anti-Human IgG4 (HRP) from Abcam (Cambridge, UK) diluted 1:10,000 in Blocking solution. The reaction mixture was incubated at room temperature for 30 min. Subsequent steps were the same as for the peptide ELISA. The cut-off for positivity on blank-corrected OD values was set at 0.10. Using that cut-off, the ELISA had a sensitivity of 58.6% and a specificity of 100% based on a set of samples from 99 nodule-positive individuals from Ghana and 9 healthy western controls. These performance characteristics are in line with other Ov16 IgG4 tests [8–15].

### Statistical analysis

To compare the antibody responses between different groups, Kruskal-Wallis test was performed. For assessment of correlation among responses, Spearman’s correlation coefficients and one-tailed *P*-value were calculated. In order to investigate trends in seropositivity over different age groups, a Chi-square test for trend was calculated. To determine the overrepresentation of prevalence in a certain group, contingency tables were prepared, and Chi-square test was performed. All analyses were performed using GraphPad Prism 7.

## Results

In total, serum samples of 303 subjects were investigated in this study including 187 children and 116 adults. Samples were selected based on their STH infection status, as determined by coprological examination. A total of 47 (17.5%) individuals were *Ascaris lumbricoides* single infected, 61 (20.1%) were *Trichuris trichiura* single infected, 15 (5.0%) were single hookworm infected, 5 (1.7%) were single *S. mansoni* infected, and 79 (22.4%) were infected with more than one helminth species (mixed infection). Additionally, 96 (31.7%) samples were included from individuals without patent infection with STH or *S. mansoni* (Table 1 and Additional file 1: Table S1).

**Fig. 1:**
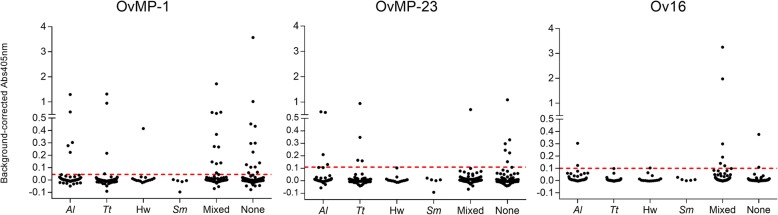
ELISA measurements of the antibody responses to OvMP-1 (IgG), OvMP-23 (IgG) and Ov16 (IgG4) in sera from study participants from Jimma Town (Ethiopia). Participants are grouped according to the type of helminth infection that was detected by coprological examination. The dashed lines indicate the antigen-specific cut-offs: 0.045 for OvMP-1; 0.11 for OvMP-23 and 0.10 for Ov16. *Abbreviations*: *Al*: *A. lumbricoides*; *Tt*: *Trichuris trichiuris*; Hw: Hookworm; *Sm*: *Schistosoma mansoni*

Total IgG against OvMP-1 and OvMP-23 and IgG4 levels against Ov16 were measured by ELISA. It was found that 34 (11.2%) samples were positive for OvMP-1, 15 (5.0%) for OvMP-23, and 11 (3.6%) for Ov16 IgG4 (Fig. 1). To investigate whether seroprevalence for either OvMP-1, OvMP-23 or Ov16 was linked to having a specific patent infection, samples were grouped according to known helminth infection. No significant difference in seroprevalence could be detected among the different groups for either of the three ELISA’s, as based on Chi-square test (OvMP-1: *χ*^2^ = 3.39, *df* = 5, *P* = 0.64; OvMP-23: *χ*^2^ = 5.266, *df* = 5, *P =* 0.38; and Ov16: *χ*^2^ = 7.14, *df* = 5, *P =* 0.21). Also, when grouping all samples from individuals with any patent STH and/or *S. mansoni* infection and comparing them with samples from individuals without any patent infection, no significant difference was found (OvMP-1: *χ*^2^ = 0.7596, *df* = 1, *P* = 0.38; OvMP-23: *χ*^2^ = 0.5043, *df* = 1, *P =* 0.48 and Ov16: : *χ*^2^ = 0.9613, *df* = 1, *P =* 0.33). Moreover, the intensity of the response to OvMP-1 or OvMP-23 was not significantly stronger in individuals carrying patent STH or *S. mansoni* infections compared to individuals without a patent infection (Fig. 1). For Ov16 IgG4, there was a significant difference in the intensity of the response between the different groups (Kruskal-Wallis test, *P* < 0.0001). However, due to the limited number of positive samples (11 out of 303), these results should be interpreted with some reservation.

In total, 33 out of 34 individuals (97.1 %) that tested positive for OvMP-1 and 14 out of 15 individuals (93.3 %) that tested positive for OvMP-23 were Ov16 negative. To determine whether the reactivity to OvMP-1 or OvMP-23 in Ov16 negative individuals was affected by the presence of STH or *S. mansoni* infection, Chi-square tests were performed. These tests showed that there was no significant effect of infection with STH or *S. mansoni* on providing a positive result on OvMP-1 or OvMP-23 peptide ELISA in Ov16 negative individuals (STH: *χ*^2^ = 0.5004, *df* = 1, *P* = 0.48 and *χ*^2^ = 0.526, *df* = 1, *P* = 0.47 and *S. mansoni*: *χ*^2^ = 0.7509, *df* = 1, *P* = 0.39 and *χ*^2^ = 1.317, *df* = 1, *P* = 0.25, respectively).

Although *S. mansoni* eggs were detected in the stool of 26 individuals (8.6%), 21 of these individuals were harboring at least one other helminth species and were thus included in the mixed infection group. From all 26 *S. mansoni* infected individuals, 4 were positive for OvMP-1 antibodies. None of these 4 individuals were infected with only *S. mansoni* and all were negative for Ov16 IgG4. In addition, none of the 26 *S. mansoni* infected individuals had antibodies to OvMP-23.

No correlation was seen between the results obtained from the Ov16 IgG4 and OvMP-1 or OvMP-23 IgG ELISAs. A mutual correlation was observed between the two peptide serology markers (Spearman’s *r* = 0.478, *P* < 0.0001). However, this correlation is mainly driven by the high number of samples that were negative for both markers (259 out of 303) but which showed correlating background signals. This positive correlation disappears if the analysis is performed only including samples that were positive for one of both peptide markers.

**Fig. 2:**
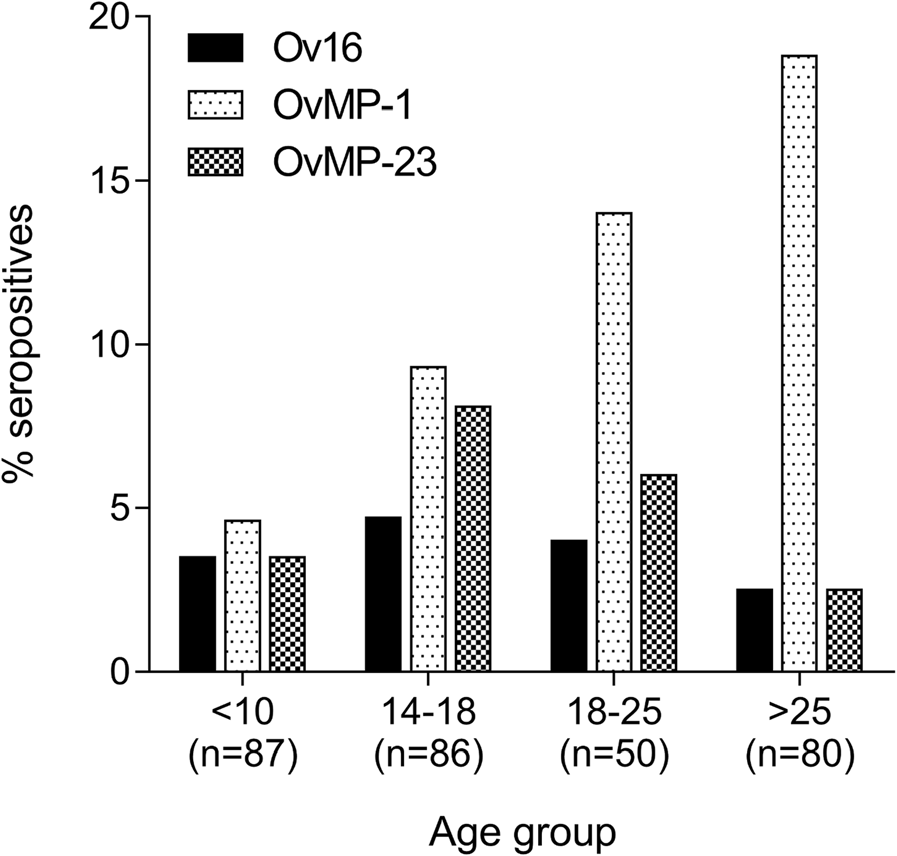
The percentage of seropositive individuals for OvMP-1, OvMP23 and Ov16 according to age group

When individuals were grouped according to different age categories (< 10, 14–17, 18–24 and > 24 years), a significant increase in seropositivity for OvMP-1 can be observed in increasing age groups (Chi-square test for trend: *χ*^2^ = 9.089, *df* = 1, *P* = 0.0026), while for OvMP-23 and Ov16 IgG4 no significant differences (Chi-square test for trend: *χ*^2^ = 0.2279, *df* = 1, *P* = 0.63 and *χ*^2^ = 0.1508, *df* = 1, *P* = 0.70, respectively) were observed (Fig. 2). Although it might appear that for Ov16 and OvMP-23 seropositivity first increases and from the age of 18 onwards decreases, this trend is not significant. This might be due to the very low number of seropositive samples in each age group. In the group of children under 10 years of age (*n* = 87), only 3 were positive for Ov16 IgG4. Moreover, the respective OD values of these samples were between 0.1 and 0.2, which is barely across the selected cut-off value for the Ov16 ELISA. In this same age category, 4 (4.6%) tested positive for OvMP-1 and 3 (3.5%) tested positive for OvMP-23 antibodies.

## Discussion

The assessment of cross-reactivity with other related and/or co-endemic infectious agents is a critical part of the analytical validation of novel diagnostic tools. Hence, this work represents an essential part of the analytical validation of the serological markers OvMP-1 and OvMP-23. We investigated the prevalence of antibodies against *O. volvulus* antigens in Jimma Town, an urban setting in the southwestern part of Ethiopia. Ethiopia is a country that has been identified as endemic for *O. volvulus* for many years [19]. Jimma Town itself is considered hypo-endemic for onchocerciasis. Using the anti-Ov16 IgG4 ELISA, exposure to *Onchocerca* was determined for all individuals (*n* = 303). Using this standard assay, the overall seropositivity rate for *O. volvulus* in our study population was 3.6%, which suggests a hypo-endemic transmission setting [6]. When focusing on the children under 10 years of age, a seroprevalence of 3.5% was observed. This finding indicates low prevalence of *O. volvulus* exposure but is still considerably higher than the target seroprevalence of 0.1% in the age group under 10 years as set by the WHO to define end of transmission and elimination [14, 27]. This is also still above the threshold of 1% or even 2%, which has recently been proposed as a more feasible threshold with the current Ov16 based tools [16, 17]. However, it is of importance to note that the Ov16 ELISA used in this study is a research-use only test that has not yet been subjected to a cross-validation with the original Ov16 ELISA or lateral flow assays. It remains possible that the use of slightly different technology led to different seropositivity rates.

Using the newly identified serodiagnostic peptide antigens OvMP-1 and OvMP-23, respectively 11.2% and 5.0% of investigated individuals tested positive. As described before, both peptide ELISAs were not 100% specific for *O. volvulus* and also appeared to provide a positive signal in individuals that were infected with *Brugia malayi*, *Wuchereria bancrofti* or STHs [18]. Of the samples that were seropositive for OvMP-1 (*n* = 34) or OvMP-23 (*n* = 15), 97.1% and 93.3%, respectively, were seronegative for anti-Ov16 IgG4 antibodies. However, these samples were not correlated to having a patent infection for STH or *S. mansoni*, indicating that carrying a patent STH or *S. mansoni* infection does not increase the chances of having antibodies that react with either OvMP-1 or OvMP-23. These individuals were therefore either truly exposed to *O. volvulus* but missed by the Ov16 IgG4 ELISA or infected with other agents that cause cross-reactivity with these peptides.

Based on the work presented here, the reactivity that was previously observed to OvMP-1 in STH infected individuals from Flores (Indonesia) is likely caused by an immunological agent other than STH or *Schistosomes* [18]. For OvMP-23 the data presented here confirm the previous observations that STH infected individuals do not have measurable antibody responses to the peptide [18].

Interestingly, in this area that is hypo-endemic for *Onchocerca*, it appeared that the peptide serology markers OvMP-1 and OvMP-23 did not mutually correlate, nor did they correlate with the Ov16 IgG4. It is not clear what the underlying cause is for this absence of correlation. Since no true gold standard diagnostic test exists for infection with *O. volvulus*, it is difficult to draw conclusions about the samples that have discordant results for these serological markers. It is possible that recognition of the three markers is affected by different life-cycle stages or sex of the present parasite. Alternatively, this might also reflect individual differences in MHC Class II haplotypes [28, 29]. A combination of the serological markers might be needed to properly define infection status. Similar observations were also made in a study where a set of four *O. volvulus* recombinant proteins were evaluated as serological markers, and where correlation between these markers was also often very weak [30]. One explanation for the lack of correlation between Ov16 IgG4 and the peptide markers might be found in the fact that it takes on average 15 months before an Ov16 IgG4 response can be detected, while an IgG response might already be observed 16 weeks after exposure [31, 32]. While this is a drawback of IgG4-based tests, it has the advantage of showing very high specificity [33].

While Ov16 seroprevalence was low over all the age groups, for OvMP-1 there appeared to be a significant trend towards increased seroprevalence as age increases. This type of trend is typically attributed to ongoing transmission settings where development of antibodies is slow and prevalence is higher in the adult population [34]. For OvMP-23 there also appeared to be an increase in the age group between 14 and 18 years, which would indicate ongoing transmission. However, seroprevalence for OvMP-23 decreased again with increasing age, although not significantly. It is well known that immune responses against certain antigens can be shorter lived than responses against other antigens [35, 36]. Therefore, this pattern might be indicative of the shorter longevity of OvMP-23 antibodies, resulting in a stabilization or even reduction in seroprevalence in higher age groups. These patterns might however also indicate that both Ov16 and OvMP-23 are specifically related to *O. volvulus* for which transmission in the studied area is low or even interrupted. In addition to exposure to *O. volvulus*, antibody reactivity to OvMP-1 and OvMP-23 might also be stimulated by other, yet undefined agents or organisms. Future evaluations with these diagnostic peptides are required to help elucidate the possible origin of cross-reactive signals.

## Conclusions

This work demonstrates that individuals with patent STH or *S. mansoni* infections have no higher prevalence of antibodies to both OvMP-1 and OvMP23 onchocerciasis peptide markers compared to uninfected individuals. This is an important aspect in the analytical validation of these biomarkers. However, more work is needed to evaluate the clinical utility of the selected peptide ELISAs in *Onchocerca* endemic and non-endemic populations, and to further investigate the origin of the discordancy with the Ov16 ELISA test and their mutual discordancy.

## Additional file

Additional file 1: **Table S1.** The collected coprological and serological data of all evaluated samples. (XLSX 156 kb)

## Abbreviations

APOC: African Programme for Onchocerciasis Control
ELISA: Enzyme-linked immunosorbent assay
LF: Lymphatic filariasis
MDA: Mass drug administration
MHC: Major Histocompatibility Complex
RDT: Rapid diagnostic test
REMO: Rapid Epidemiological Mapping of Onchocerciasis
STH: Soil-transmitted helminths
WHO: World Health Organization
